# Cortisol Responses to Naturally Occurring Psychosocial Stressors Across the Psychosis Spectrum: A Systematic Review and Meta-Analysis

**DOI:** 10.3389/fpsyt.2020.00513

**Published:** 2020-06-11

**Authors:** Alexis E. Cullen, Sushma Rai, Meghna S. Vaghani, Valeria Mondelli, Philip McGuire

**Affiliations:** ^1^Department of Psychosis Studies, Institute of Psychiatry, Psychology & Neuroscience, King’s College London, London, United Kingdom; ^2^Department of Psychological Medicine, Institute of Psychiatry, Psychology & Neuroscience, King’s College London, London, United Kingdom; ^3^NIHR Biomedical Research Centre, South London and Maudsley NHS Foundation Trust, London, United Kingdom

**Keywords:** schizophrenia, psychosis, hypothalamic-pituitary-adrenal axis, stress responsivity, cortisol, concordance, trauma, adversity

## Abstract

**Background:**

Individuals with established psychosis and those at high-risk for the disorder have been found to show abnormalities within the hypothalamic-pituitary-adrenal (HPA) axis, including elevations in basal and diurnal cortisol, but a blunted cortisol awakening response. However, the extent to which these features are associated with psychosocial stressors encountered in the natural environment (which are known to be more commonly experienced by these groups, and more distressing) is currently unclear. We therefore conducted a systematic review and meta-analysis to investigate the concordance between naturally-occurring psychosocial stressors and cortisol levels in these populations.

**Methods:**

PubMed, PsycINFO, and EMBASE were searched up to November 2019 to identify studies examining the concordance between psychosocial stressors and cortisol in healthy controls and individuals on the psychosis spectrum (patients with established psychosis and/or high-risk individuals). An overall meta-analysis (including data for all stressor-cortisol pairings) was performed to determine the degree of concordance irrespective of group status, with meta-regression employed to test whether the degree of concordance differed in established psychosis and high-risk groups compared to controls. Planned stratified analyses were then performed to examine group differences (where established psychosis and high-risk groups were combined) within individual stressor-cortisol pairings.

**Results:**

Eighteen studies (16 datasets) were eligible for inclusion. The overall model, comprising 134 effect sizes, showed that stressors and cortisol measures were only weakly correlated [*r=*0.05 (95% CI: -0.00 to 0.10), *p*=0.059] and that neither established psychosis status (*r=*0.01, *p*=0.838) nor high-risk status (*r=*0.02, *p*=0.477) had a significant effect of the strength of correlation. In stratified analyses, significant differences between healthy controls and psychosis spectrum groups were observed for only one of the six stressor-cortisol pairings examined, where life event exposure and diurnal cortisol were positively correlated in controls [*r=*0.25 (95% CI: 0.01 to 0.46)], but negatively correlated in the psychosis spectrum group [*r=*-0.28 (95% CI: -0.49 to -0.04)].

**Conclusions:**

Overall, we observed poor concordance between naturally-occurring psychosocial stressors and cortisol irrespective of stressor type, cortisol measure, or group status. We consider a range of methodological factors that may have obscured the ability to detect “true” associations and provide recommendations for future studies in this field.

## Introduction

Research conducted over the past four decades has provided evidence to suggest that psychosocial stress contributes to the onset and exacerbation of psychosis. Meta-analyses indicate that major life events and childhood trauma (typically encompassing experiences of neglect and abuse) are associated with increased risk of developing psychotic disorders (1, 2). Furthermore, in patients with established psychosis, minor daily stressors have been associated with psychotic symptom intensity (3–6) and illness relapse (7, 8). More recently, focus has shifted to individuals identified as being at increased risk for psychosis by virtue of a family history (FHx) of illness and/or clinical features, the latter including individuals who fulfil ultra-high risk (UHR) criteria, present with schizotypal personality traits, or report psychotic experiences (PEs). Studying these populations overcomes some of the potential confounds that often arise in studies of patients with established psychosis (e.g., retrospective recall, antipsychotic medication, and stress associated with illness onset). Such studies demonstrate that high-risk individuals are also more frequently exposed to childhood trauma, major life events, and minor daily stressors, experience greater distress in relation to these events, and report higher levels of perceived stress compared to their healthy peers (4, 9–17). Although these studies lend support to the notion that stress may play a causal role in the development of psychosis, the biological mechanisms underlying this relationship remain unclear.

One leading hypothesis, the neural diathesis-stress model (18–20), proposes that the hypothalamic-pituitary-adrenal (HPA) axis plays a major role in mediating the effects of stress on psychosis development. Specifically, it is hypothesized that individuals with increased vulnerability for psychosis are more sensitive to the effects of psychosocial stress due to abnormalities within the HPA axis (e.g., HPA hyperactivity/dysregulation or increased glucocorticoid sensitivity) and that these HPA axis abnormalities in turn trigger the onset of psychosis by acting on dopaminergic and glutamatergic pathways (20). The model is supported by individual studies and meta-analyses reporting elevations in basal and diurnal cortisol (21–28), a blunted cortisol awakening response [CAR (23, 29–31)], and enlarged pituitary volume (23, 32, 33) among high-risk individuals and psychosis patients. It is important to note that these measures represent different attributes of HPA axis function: While the increases in basal/diurnal cortisol levels and pituitary volume likely reflect chronic hyper-activation of the HPA axis, it is thought that the CAR is a distinct HPA axis component driven by endogenous processes, possibly related to anticipation of the demands of the upcoming day (34, 35). Together, these findings imply that HPA axis dysfunction characterizes individuals on the psychosis spectrum; however, evidence linking these HPA axis changes to psychosocial stressors is lacking.

Several systematic reviews/meta-analyses have been published concerning the “stress response” in psychosis [for an overview see (36)]; however, the majority have considered HPA axis abnormalities and psychosocial stressors in isolation rather than the concordance (i.e., degree of association) between these measures. Of those that specifically examined HPA axis responsivity to stress (37–39) all three looked exclusively at responses to acute psychosocial stressor tasks, concurring that individuals with schizophrenia and psychosis show a blunted cortisol response relative to healthy controls. While recent work indicates that laboratory-based psychosocial stressor tasks can be considered ecologically valid [i.e., associations observed between cortisol responses to these tasks and responses to real-world examination stress (40)] these “performance-related” stressors likely differ in both nature and frequency to the stressors shown to be etiologically relevant to psychosis (e.g., major life events and childhood trauma). Understanding the concordance between psychosocial stressors encountered in the natural environment and HPA axis function is important for several reasons: If psychosocial stressors are found to correlate with HPA axis markers, then this provides a plausible biological mechanism for how stress might contribute to the onset and maintenance of psychosis, further strengthening the case for this being a causal factor. Similarly, if high concordance between these measures is found, then this supports the notion that the HPA axis abnormalities observed among individuals on the psychosis spectrum are driven by psychosocial stressors, as opposed to being simply epiphenomena (perhaps indicative of global metabolic abnormalities). Furthermore, comparing the degree of concordance in healthy individuals and those on the psychosis spectrum will help to clarify the extent to which abnormal stress responsivity (either hyper- or hypo-responsivity) is a feature of psychosis. Such work may ultimately enable targeted interventions to be delivered to those who are more sensitive, at least biologically, to the effects of psychosocial stress.

To this end, we conducted a systematic review and meta-analysis of studies examining the concordance between naturally-occurring psychosocial stressors and HPA axis function among individuals on the psychosis spectrum. Given that cortisol is the most widely used measure of HPA axis function (18, 35), we restricted our review to studies examining stressor-cortisol concordance only. Our primary aim was to test whether the degree of concordance differed among healthy individuals and those on the psychosis spectrum (patients with established psychosis and high-risk individuals). Meta-analytic evidence indicates that the degree and direction of concordance varies across different cortisol measures and stressor types; for example, chronic stress has been found to correlate positively with overall diurnal output, afternoon/evening levels, and the CARi (increase in cortisol following awakening) but negatively with basal morning levels (41, 42). We were therefore concerned that combining all effect sizes in a single analysis could lead to a neutral effect overall. To mitigate against this, in addition to performing an overall meta-analysis (which included all effect sizes), we also conducted analyses within individual stressor-cortisol pairings.

**Figure 1:**
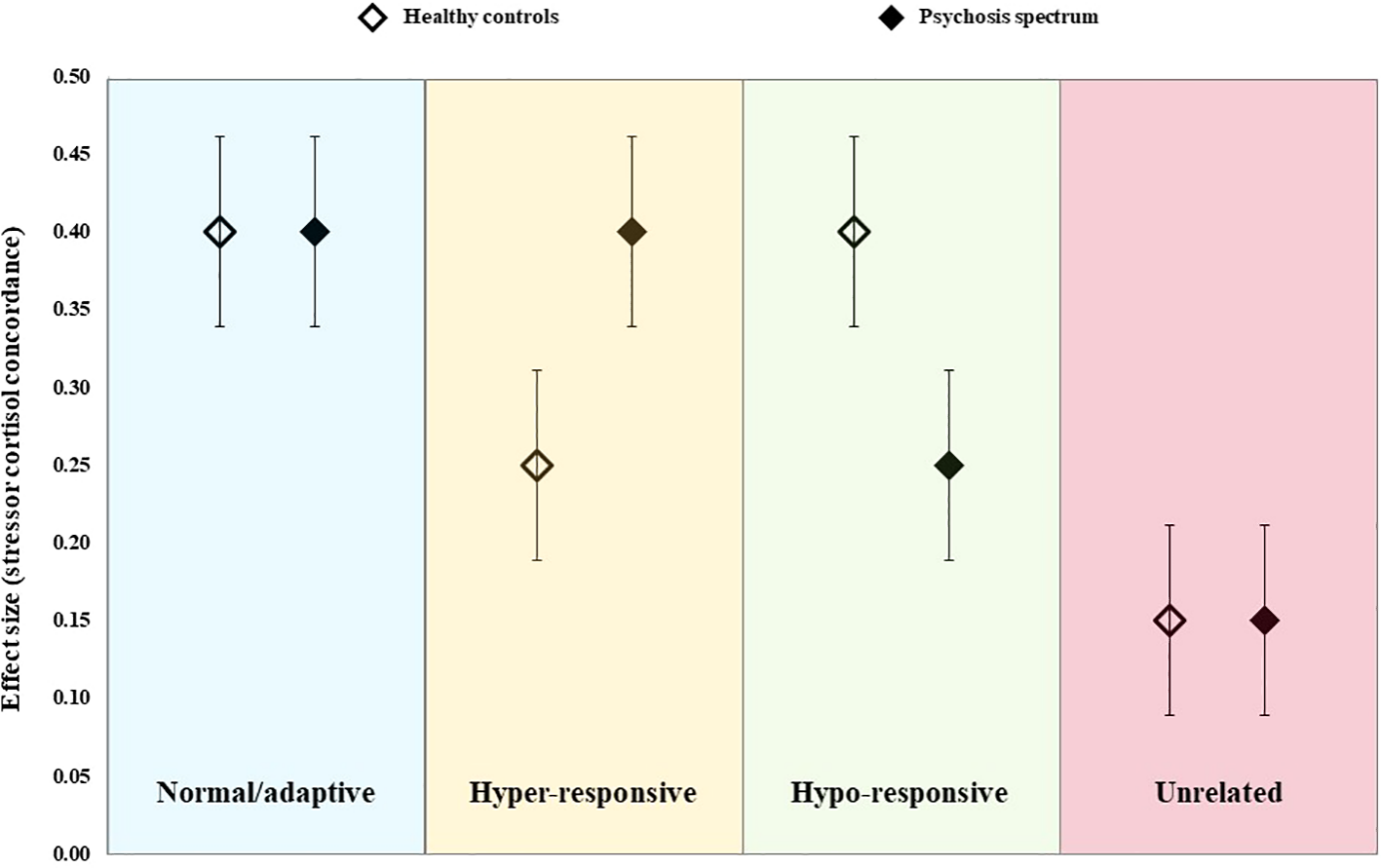
Schematic representation of the four alternative hypotheses tested in the current meta-analysis.

As this was the first review to address these specific questions, we tested four possible (and competing) hypotheses regarding the pattern of findings across controls and psychosis spectrum groups (see **Figure 1**). The “normal/adaptive” hypothesis (blue) proposes that the degree of concordance between naturally-occurring psychosocial stressors and cortisol is moderate-to-strong in both healthy and psychosis spectrum individuals, but that there is no difference in the degree of concordance across groups. If supported, this would imply that the HPA axis abnormalities observed among psychosis spectrum groups reflects a normal/adaptive response to the high levels of psychosocial stress experienced by this population, thus, the HPA axis itself is responding to stress appropriately. The “hyper-responsive” hypothesis (yellow) proposes that psychosocial stressors will be associated with cortisol in both groups, but that this relationship will be stronger among those on the psychosis spectrum. Support for this hypothesis would suggest that psychosocial stressors measured in concordance studies are, at least partially, responsible for the HPA axis abnormalities in psychosis spectrum individuals, but that the HPA axis responds excessively to these stressors in this population. The reverse situation is represented by the “hypo-responsivity” hypothesis (green), whereby the degree of concordance is moderate-to-high in controls but is blunted (perhaps due to glucocorticoid sensitization) in psychosis spectrum groups. If supported, this would suggest any HPA axis abnormalities observed in the psychosis spectrum group occur despite the fact that this group experiences greater psychosocial stress exposure/distress. Alternatively, the pattern observed may be that presented in the “unrelated” hypothesis (red), whereby concordance in both groups is similar but weak. Such findings would indicate that the psychosocial stressors commonly measured in concordance studies are unrelated to HPA axis function, implying that any cortisol abnormalities observed in psychosis spectrum groups must be driven by other factors (e.g., unmeasured stressors, genetic variations, or a manifestation of a globally dysregulated physiological system). However, poor concordance could also reflect measurement error (of psychosocial stressors, cortisol levels, or both). These competing hypotheses were tested statistically by comparing pooled effect sizes in psychosis spectrum and healthy control groups.

## Methods

The protocol for this systematic review and meta-analysis was registered prospectively on PROSPERO (CRD42019159290), the search strategy and reporting was conducted in compliance with the Meta-Analysis of Observational Studies in Epidemiology (MOOSE) guidelines (43).

### Search Strategy

PubMed and Ovid (PsycINFO, EMBASE) databases were searched for articles published up to November 2019 using the following terms: (genetic high risk) OR family history) OR prodrom*) OR at risk mental state) OR clinical high risk) OR ultra high risk) OR sibling*) OR offspring*) OR relative*) AND (psychotic) OR psychosis) OR schizophren*) OR schizotyp*) OR psychotic experiences) OR subclinical psycho*) OR psychotic) OR psychosis) OR schizophren*) OR schizotyp*) OR psychotic experiences) OR subclinical psycho*) AND (trauma) OR advers*) OR neglect) OR stress*) OR hassles) OR life events) OR maltreatment) OR abuse) AND (HPA axis) OR stress response) OR cortisol) OR glucocorticoid). The searches were performed independently by two researchers (SR, MV). No restrictions were applied for year of publication. Reference lists of eligible studies and relevant reviews were manually searched to identify additional studies.

### Study Selection

We included observational studies (cross-sectional, case-control, and cohort studies) that examined the relationship between naturally-occurring psychosocial stressors and cortisol in individuals on the psychosis spectrum (patients with established psychosis or those at elevated risk for psychosis) and healthy controls. Patients with diagnoses of first-episode psychosis, multi-episode psychosis, schizophrenia, and schizoaffective disorder were eligible for the established psychosis group. Consistent with a previous review by our group (33), we defined high-risk individuals as those who met criteria for one of the following groups: i) “ultra-high risk” for psychosis [also known as “clinical high-risk” or an “at-risk mental state” (44, 45)] as determined using a well-defined assessment tool, ii) family history (FHx), as conferred by a first- or second-degree relative with psychosis, iii) schizotypal personality disorder (SPD) or high scores on a schizotypal personality checklist, or iv) presence of psychotic-experiences (PEs: also known as psychotic-like experiences or subclinical psychotic symptoms).

All articles identified in the search were independently rated for eligibility by two authors (SR, MV). Disagreements were resolved following a discussion with a third author (AC). Article titles and abstracts were first screened to remove those that were clearly not relevant to the review; a full text review was then performed for all potentially eligible articles (both phases performed in duplicate). Original studies meeting the following criteria were eligible: i) inclusion of a psychosis spectrum group (established or high-risk, as defined above) and healthy control group, ii) assessment of naturally-occurring psychosocial stressors (e.g., exposure to daily stressors, life events, trauma, adversity, distress associated with these events, or perceived stress), iii) measurement of cortisol (basal, diurnal, or CAR as measured in saliva, blood, or hair), iv) concordance between psychosocial stressor and cortisol reported, and v) published in English in a peer-reviewed journal. Articles that did not include a control group or report the association between psychosocial stressors and cortisol were excluded. Conference abstracts were not included (as none included sufficient data), but where relevant abstracts were identified, additional searches (by author name) were conducted to determine whether a full text article had been subsequently published and/or corresponding authors were contacted for further details. Where studies with potentially overlapping samples were identified we contacted study authors to clarify.

### Data Extraction

Two researchers (SR, MV) extracted study characteristic data from eligible articles, this included: author(s), year of publication, psychosis spectrum group(s), psychosis spectrum group recruitment/identification method, sample size, mean age and sex of psychosis spectrum and control groups, proportion treated with antipsychotic medication, stressor measurement method, cortisol measure (tissue and type), and lapse-of-time between stress measurement and cortisol collection. Researchers were not blind to the names of authors, journals, or institutions. A third author (AC) then checked all study details for accuracy and extracted data necessary for effect size computation. The latter varied across studies and included any statistical value representing a within-group measure of the association between psychosocial stressors and cortisol (e.g., correlation coefficient, beta coefficient, or mean and standard deviation of cortisol for participants exposed and not exposed to stressor). Where these data were not provided for each group separately, we contacted study authors for additional details (46–56) which were provided in all instances.

### Assessment of Study Quality

A modified version of the Newcastle-Ottawa scale [NOS (57)], a quality appraisal tool for case-control, cohort, and cross-sectional studies, was created for the purposes of the review to capture pertinent features. The modified tool included 11 items covering three domains (selection, comparability, exposure/outcome) and was designed to be applicable to any of the above study designs (see **Supplementary Table 1** for a detailed description of the items). The maximum score available across the 11 items was 16. All studies were rated independently against these criteria by two authors (AC, SR) with disagreements resolved by discussion.

### Statistical Analyses

All computations and statistical analyses were conducted using Stata version 16 (58). In order to facilitate pooling of effect sizes (representing the association between psychosocial stressors and cortisol, in each group separately) it was necessary to first derive a common effect size for all studies. As correlational coefficients (r) were the most commonly-reported effect sizes, and are easily-interpretable [values of 0.1, 0.3, and 0.5 reflecting small, moderate, and large magnitudes of effect, respectively (Cohen, 1988)] we requested r values from study authors where these were not provided, or derived these from alternative statistics where possible. Specifically, for studies reporting means and standard deviations (SD), we first computed standardized mean differences (d), representing the difference in cortisol levels between those with and without stressor exposure, which were then converted to correlation coefficients (59). As beta coefficients (B) derived from regression models examining the effect of stressors (measured as continuous variables) on cortisol could not be converted to r values without prior standardization of variables, effect sizes from studies reporting these values (53, 60) could not be included in meta-analyses; these results were, however, retained in the systematic review. In order to perform meta-analyses, all correlation coefficients were transformed to a Fisher’s z score (59); for presentation purposes, pooled z scores and associated confidence intervals were reverse-transformed to the original units for ease of interpretation.

As nearly all studies included in the review provided multiple effect sizes derived from the same study sample, thereby violating the independence assumption, it was necessary to account for dependence of effects. For the overall meta-analysis (which included data for all stressor-cortisol pairings) we therefore used robust variance estimation (RVE) which accounts for correlated effects (61). We first derived the unconditional overall effect size (degree of concordance), irrespective of group status, by estimating the constant term only (62). This analysis was performed to determine whether the pooled correlation across all groups and studies was statistically different from zero. Next we tested the effect of group status by including two dummy variables, “established psychosis” and “high risk”, to determine whether the effect sizes (degree of concordance) in these groups differed from controls. To derive pooled effect sizes for all groups (including the control group) we then performed stratified analyses to derive the mean effect size in each group separately. Finally, in a univariate meta-regression model, we tested the effect of NOS scores on effect sizes. For all RVE models we applied a random effects weighting scheme which assumes that effect sizes from the same study are correlated with each other. The assumed value of rho was set at 0.5 after sensitivity analyses performed on the entire sample showed that there were no differences when rho values of 0.1, 0.3, 0.5, and 0.8 were applied. Heterogeneity was assessed by means of the Tau statistic (62), which provides an estimate of the standard deviation of the true effect (59). Small sample bias (i.e., publication bias) was assessed visually by means of a funnel plot but was not tested statistically due to dependence of effects.

As we anticipated that the degree and direction of concordance would vary across different cortisol measures and stressor types (41, 42), we next performed planned stratified analyses to examine group differences within individual stressor-cortisol pairings. However, as this greatly reduced the number of studies contributing to each analysis, and RVE performs poorly when the degrees of freedom are small (63), it was necessary to employ a different approach to deal with dependent effects. Thus, for studies that included more than one psychosis spectrum group (for example, an established psychosis group and a high-risk group), we first computed within-study pooled effect sizes for each stressor-cortisol pairing, which combined data from all psychosis spectrum groups. As such, each study contributed only two effect sizes to each stressor-cortisol pairing: one for the control group, and the other a pooled effect size derived from all psychosis spectrum groups. Stratified analyses were performed when three or more studies were available using the default settings within Stata 16 (random effects model with restricted maximum likelihood weighting applied). We used the subgroup command which enables the derivation of subgroup specific pooled effect sizes (and heterogeneity estimates) and a between-group comparison of effect sizes. Statistical significance for all analyses was set at p < 0.05 (two-tailed). Heterogeneity was assessed *via* the Cochran Q and the I² statistics, where classification of the latter as likely unimportant (0%–40%), moderate (30%–60%), substantial (50%–90%), or considerable (75%–100%) is dependent on the magnitude and/or direction of effects and statistical significance (Cochran Q) of heterogeneity (64).

## Results

### Search Results

**Figure 2:**
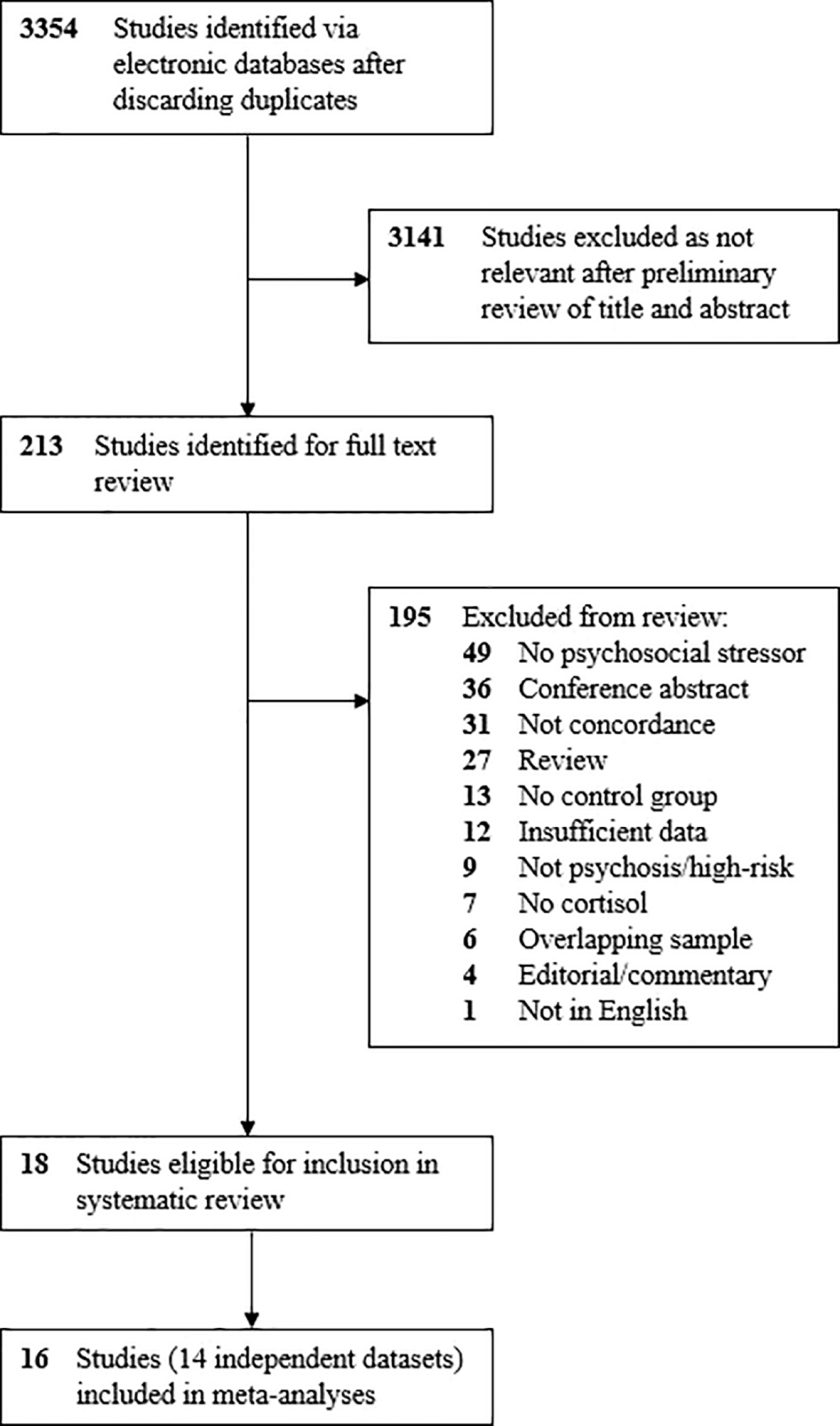
Search process.

After removing duplicates, 3,354 studies were identified in the initial search (**Figure 2**). Of these, 3,141 were excluded following a preliminary review of the title and abstract, with a full-text review performed for 213 articles. After screening studies for eligibility, 18 met criteria for inclusion in the review and meta-analysis (31, 46–56, 60, 65–69), all of which were published in the last decade. Details of the 18 studies are provided in **Table 1**.

**Table 1:** Characteristics of studies included in systematic review and meta-analyses.

Author	Group	*N*	Age^1^	% Male	Stress type (measure)	Cortisol measures
Aas et al. (69)	SCZ HC	28 94	33.6 35.3	54% 51%	Childhood trauma (CTQ)	Hair (3 cm)
Ciufolini et al. (46)	FEP HC	169 133	28.1 26.9	65% 36%	Childhood trauma (CECA)	Saliva (CAR and diurnal)
Collip et al. (60)^2^	FHx HC	60 63	28.8 33.3	37% 29%	Daily event stress (ESM)	Saliva (ESM)
Cullen (31)	PE FHx HC	33 22 40	12.8 13.3 13.1	70% 50% 43%	Negative life events; Daily hassles	Saliva (CAR and diurnal)
Faravelli et al. (67)	PD HC	54 102	43.7 43.5	56% 52%	Childhood trauma (CECA)	Saliva (basal morning and evening)
Garner et al. (68)	FEP HC	39 25	20.6 22.5	67% 84%	Perceived stress (PSS)	Serum (basal morning)
Heinze et al. (2015)	UHR^+1a^ HC	30 28	21.0 20.0	13% 7%	Perceived stress (PSS), Childhood trauma (CTQ)	Hair (3 cm)
Hirt et al. (55)	UHR ESCZ CSCZ HC	29 34 24 38	22.5 24.0 35.5 24.0	79% 65% 79% 63%	Childhood trauma (MACE)	Hair (3 cm)
Labad et al. (50)^3^	UHR HC	39 44	22.3 23.2	69% 65%	Perceived stress (PSS); Stressful life events (HRSS)	Saliva (basal morning and CAR); Serum (basal morning)
Labad et al. (48)^3^	UHR FEP HC	21 34 34	22.1 23.9 24.3	71% 71% 71%	Perceived stress (PSS); Stressful life events (HRSS)	Saliva (CAR and diurnal slope)
Labad et al. (49)^3^	ROP HC	56 47	24.8 23.8	63% 53%	Childhood trauma (CTQ); Stressful life events (HRSS)	Saliva (CAR and diurnal slope)
Mondelli et al. (65)	FEP HC	50 36	29.2 27.3	64% 72%	Life events (BLEQ); Perceived stress (PSS); Childhood trauma (CECA)	Saliva (CAR and diurnal)
Moskow (2016)	UHR HC	348 93	15.6 15.2	56% 65%	Daily stress (DSI)	Saliva (basal morning)
Nordholm et al. (51)	UHR FEP HC	41 40 46	23.9 24.1 24.7	43% 55% 58%	Perceived stress (PSS); Life events (BLEQ)	Saliva (CAR and diurnal)
Seidenfaden et al. (52)	SCZ HC	37 39	32.3 31.7	46% 51%	Childhood trauma (CATS); Perceived stress (PSS)	Plasma (basal morning); Saliva (diurnal)
Soder et al. (56)	PE FHx HC	43 32 35	26.2 33.3 27.3	33% 31% 37%	SES; Migration; Minority status; Perceived discrimination; Social undermining; Ostracism experience; Child abuse; Bullying victimization; Trauma	Hair (3 cm)
Streit et al. (54)	SCZ HC	159 82	40.3 32.9	36% 40%	Perceived stress (SSCS)	Hair (3 cm)
Vaessen et al. (53)^2^	FHx PD HC	47 73 67	42.9 43.8 39.9	36% 55% 52%	Daily event stress (ESM)	Saliva (ESM)

^1^Mean age in years; ^2^ Data from these studies are not included in meta-analyses as a common effect size could not be derived from these studies; ^3^ Due to partially-overlapping samples and measures, the corresponding author provided a single dataset comprising the largest study groups which is used in all subsequent analyses in this review (70). FEP, first-episode psychosis; HC, healthy control; PE, psychotic experiences; FHx, family history of psychosis; SCZ, schizophrenia; UHR, ultra-high risk; UHR^+1a,^ group includes help-seeking youth meeting UHR (stage 1b) and stage 1a criteria; ESCZ, early-stage schizophrenia; CSZC, chronic schizophrenia; ROP, recent-onset psychosis; PD, psychotic disorders; CECA, Childhood Experience of Care and Abuse Questionnaire; ESM, experience sampling method; PSS, perceived stress scale; MACE, Maltreatment and Abuse Chronology of Exposure; HRSS, Holmes-Rahe Social Readjustment Scale; CTQ, Childhood Trauma Questionnaire; BLEQ, Brief Life Events questionnaire; DSI, Daily Stress Inventory; CATS, Child Abuse and Trauma Scale; SES, socioeconomic status; CAR, cortisol awakening response.

Partially overlapping samples were identified for five studies. Three studies authored by Labad and colleagues (48–50) included overlapping study groups and stressor/cortisol measures. The corresponding author provided a combined dataset that included data for the largest available UHR, first-episode psychosis (FEP), and healthy control subgroups which was used for all meta-analyses (studies retained as separate when describing characteristics). The combined dataset is herein referred to as (70). Two studies included participants from the UK Genetic and Psychosis (GAP) study (46, 65); as both examined the association between cortisol and childhood trauma, we used the largest sample for this analysis (46); however, the earlier study (smaller sample) was retained as it included additional stress measures not examined in the later study.

### Study Characteristics

#### Group Status and Psychosis Spectrum Definitions

As dictated by our inclusion criteria, all studies included a healthy control group and at least one psychosis spectrum group. Four studies included both high-risk and established psychosis subgroups (48, 51, 53, 55), eight included established psychosis groups only (46, 49, 52, 54, 65, 67–69), and six examined high-risk groups only (31, 47, 50, 56, 60, 66). The most commonly-examined established psychosis subgroup was FEP (n=5), a further study examined recent-onset psychosis (49) which included FEP patients, two studies included patients with psychotic disorder where the stage of illness was not indicated (53, 67), three studies included patients with schizophrenia (52, 54, 69), while a further study distinguished between patients with early and chronic schizophrenia (55). With regards to high-risk groups, these were most commonly youth meeting UHR criteria (n=5) and individuals with a FHx of psychosis/schizophrenia (n=4). One of the five studies examining young people at UHR also included those who presented with clinical stage 1a symptoms (47). Soder and colleagues included a high-risk group comprising individuals who scored above threshold on a measure of psychotic experiences (PEs), whilst a further study (31) included children who at age 9–12 years presented with PEs in combination with other antecedents of schizophrenia.

#### Sample Sizes and Demographic Characteristics

The total number of healthy controls, established psychosis patients, and high-risk individuals was N=1046, N=797, and N=745, respectively. Control groups varied in size, ranging from 25 (68) to 133 (46), the latter study also comprised the largest established psychosis group (n=169). With regards to high-risk group sizes, the smallest comprised of 21 UHR individuals (48) with the largest including 348 UHR youth from the NAPLS-2 study (66). Participants in the high-risk groups were the youngest on average (mean age = 23.7 years; range: 12.8 to 42.9 years), followed by healthy controls (mean age = 27.2 years; range 13.1 to 43.5 years), with the oldest being those with established psychosis (mean age = 31.1 years; range 20.6 to 43.8 years). When averaged across studies, the percentage male was broadly similar across healthy control, established psychosis, and high-risk groups (52%, 60%, and 49%, respectively); however, this varied substantially across studies, from as low as 7% in the control group of one study (47) to 84% in the control group of another (68).

#### Psychosocial Stress Measures

Perceived stress and childhood trauma were the most common types of psychosocial stressor examined across studies (n=8 for both), followed by life event exposure (n=6). There was consistency across studies in the measures of perceived stress employed, with most studies using the Perceived Stress Scale (71). For childhood trauma, there was less consistency, with the most commonly-used measures being the Childhood Experience of Care and Abuse (CECA) questionnaire (72) and the Childhood Trauma Questionnaire (73). Daily stressors were examined in two studies (albeit using different measures), one reported both exposure and distress scores separately (31), whilst the other reported a single score that accounted for both exposure and associated distress (66); the former study also reported distress scores (both current distress and distress at the time of the event) for negative life events. The experience sampling method (ESM), a structured diary technique in which participants are prompted at multiple time-points throughout the day to report the extent to which their current activity is stressful, was used in two studies (53, 60). One study examined nine individual psychosocial stressors (56), including, socioeconomic status, migration, minority status, perceived discrimination, social undermining, ostracism experience, bullying victimization, childhood abuse, and trauma experiences.

#### Cortisol Measures

Across the 18 studies, cortisol was most frequently measured in saliva (n=12), two of these studies also examined cortisol in blood samples [serum (50); plasma (52)] with a further study examining serum only (68). Hair sampling was the second most common method used to determine cortisol levels (n=5), with all studies obtaining at least one 3 cm segment for analysis (47, 54–56, 69). With regards to the timing of cortisol collection, basal samples (saliva, plasma, and serum) were the most commonly-examined, with four studies obtaining a single measure (50, 52, 67, 68), typically in the morning, and a further study deriving a mean cortisol value from three samples obtained at 1-h intervals (66). The cortisol awakening response (CAR) was measured in saliva in seven studies (31, 46, 48–51, 65): All of these studies computed the area-under-the-curve with respect to increase (AUCi) which captures the increase in cortisol from awakening levels; one of these studies (46) also calculated the AUC with respect to ground (AUCg) representing the total amount of cortisol secreted in the hour following awakening. Diurnal cortisol was examined in saliva in six studies, five of which calculated the total cortisol output over the entire day using the AUCg (31, 46, 51, 52, 65), with the remaining study calculating the diurnal slope between samples collected at awakening and late evening (49). Two studies used the ESM method to obtain multiple salivary cortisol samples throughout the day (53, 60) with repeated observations handled using multilevel (hierarchical) models.

#### Quality Assessment

Study quality scores are presented in **Table 2**. Total scores ranged from 6 to 12 (max=16) with an average score of 8 across the 18 studies. Sample size was a concern for most studies; only three were awarded a single point for this item and none were awarded two points. Of the three studies obtaining a single point, two (46, 66) included at least 85 participants in each group and so were sufficiently large to detect a moderate correlation with 80% power at the 0.05 level. Only one study conducted an *a priori* power calculation (56); however, this was used to determine the total sample size (comprising controls, FHx, and PE groups) and so each individual group did not meet the criteria outlined above (n≥85). With regards to participants, all studies used an adequate/validated measure to confirm diagnosis (established psychosis) or high-risk status; however, only 11 studies applied the same measures to the healthy control group to confirm that these participants were free from psychotic disorder and/or did not meet high-risk criteria. A major area of weakness across the studies was the extent to which psychosis spectrum and control groups were representative/unbiased. In general, very few details were available to be able to assess the extent to which patients with established psychosis and at-risk groups were representative of the target populations, and none reported that patients were randomly selected from a registry. However, two studies reported that they attempted to recruit all patients who were newly admitted to psychiatric services operating within a large catchment area (65, 67) and so were awarded a point for this item. Similarly, details of methods used to identify and recruit controls were minimal in most studies, with only one study (60) reporting that controls were selected through random mailings to addresses in the residential areas of patients and siblings. Only three studies (31, 47, 52) reported the response rate (proportion of individuals approached who agreed to participate) for any group. One strength was that all studies either deliberately matched psychosis spectrum and control groups on age and/or sex (two points) or compared groups on these characteristics (one point).

**Table 2:** Study quality ratings with regards to assessment of stressor-cortisol concordance.

Study	Sample size adequate/determined *a priori* (max 2)	Psychosis spectrum definition valid (max 1)	Psychosis spectrum cases unbiased (max 1)	Control group unbiased (max 1)	Control status confirmed (max 1)	Response rate reported/same in both groups (max 1)	Psychosis spectrum and control groups matched (max 2)	Stress measure reliable/valid (max 2)	Cortisol measure reliable/valid (max 2)	Lapse of time between measures reported (max 1)	Potential confounds examined (max 2)	Total score (max 16)
Aas et al. (69)	0	1	0	0	1	0	1	2	1	0	1	7
Ciufolini et al. (46)	1	1	0	0	1	0	2	1	2	0	1	9
Collip et al. (60)	0	1	0	1	1	0	2	2	2	1	2	12
Cullen et al. (31)	0	1	0	0	1	1	1	2	2	1	2	11
Faravelli et al. (67)	0	1	1	0	0	0	2	1	1	0	2	8
Garner et al. (68)	0	1	0	0	1	0	2	1	1	0	0	6
Heinze et al. (47)	0	1	0	0	0	1	2	1	2	0	2	9
Hirt et al. (55)	0	1	0	0	0	0	1	1	2	0	2	7
Labad et al. (50)	0	1	0	0	0	0	2	1	1	0	2	7
Labad et al. (48)	0	1	0	0	0	0	2	1	1	0	2	7
Labad et al. (49)	0	1	0	0	0	0	2	2	1	0	2	8
Mondelli et al. (65)	0	1	1	0	1	0	1	1	2	0	2	9
Moskow (66)	1	1	0	0	1	0	1	2	1	0	0	7
Nordholm et al. (51)	0	1	0	0	1	0	2	2	1	0	1	8
Seidenfaden et al. (52)	0	1	0	0	1	1	1	1	1	0	0	6
Soder et al. (56)	1	1	0	0	1	0	1	1	1	0	2	8
Streit et al. (54)	0	1	0	0	0	0	1	2	2	0	0	6
Vaessen et al. (53)	0	1	0	0	1	0	1	1	2	1	2	9

With regards to measures, all studies employed a widely used measure of psychosocial stress (one point), with seven reporting the reliability/validity of these measures (two points). Descriptions of the cortisol collection procedure varied from brief to very detailed, with half of the studies providing a reference for the procedure and/or assessing compliance. Only three studies reported details of the timing of cortisol collection with regards to psychosocial stress measurement; two of these studies used the ESM method, where cortisol samples were collected within 10 min of the event stress rating (53, 60), the other study reported the mean lapse-of-time between completion of stress measures and collection of cortisol samples (31). Assessment of potential confounders varied across studies, ranging from very few confounders examined (age, sex, and one other measure: n=4) to a wide range of variables that were compared across groups and/or examined in relation to cortisol/stress measures (n=11).

### Description of Stressor-Cortisol Concordance Findings Across Studies

From the 16 datasets, 139 separate effect sizes were available (124 correlation coefficients, 10 standardized mean differences converted to correlation coefficients, and five beta coefficients). Of these, 123 (88%) were not statistically significant (indicating no association between stressor and cortisol), 11 (8%) were statistically significant positive associations, and five (4%) were significant negative associations. At the study level, nine (56%) of the datasets included at least one statistically significant association (31, 46, 53, 55, 56, 60, 65, 67, 70). With regards to magnitude of effect irrespective of sign (positive or negative), after excluding the five beta coefficients (which were not standardized and therefore not comparable), 53 (40%) were negligible, 62 (47%) were small, 14 (11%) were moderate, and three (2%) were large effect sizes.

#### Basal Cortisol

Basal cortisol was examined in six studies, yielding 20 separate effect sizes (morning=18; evening=2), only three of which were statistically significant. The pattern of findings varied across studies and stressor types. In one large study of patients with psychotic disorder (67), morning salivary cortisol showed a significant positive association with childhood trauma in patients [*r=*0.29 (95% CI: 0.02 to 0.52)] that was not observed in healthy controls [*r=*-0.06 (95% CI: -0.25 to 1.33)], yet evening cortisol was significantly associated with childhood trauma in controls [*r=*0.22 (95% CI: 0.03 to 0.40)] but not patients [*r=*0.14 (95% CI: -0.13 to 0.39)]. In contrast, Labad and colleagues (70), who assessed morning basal cortisol in plasma, observed no relationship with childhood trauma, instead finding a significant negative relationship with stressful life events in controls [*r=*-0.30 (95% CI: -0.54 to -0.01)] that was not present in either UHR individuals [*r=*-0.01 (95% CI: -0.34 to 0.33)] or FEP patients [*r=*0.14 (95% CI: -0.20 to 0.45)]. Plasma morning cortisol was not, however, associated with childhood trauma in either controls or individuals with schizophrenia in a further study (52). One consistent finding was that basal morning cortisol was not significantly associated with perceived stress in any group (52, 68, 70); moreover, no relationship was found between basal cortisol and daily stressors in UHR youth and healthy controls (66).

#### Cortisol Awakening Response (CAR)

In total, 37 individual effect sizes were available for the CAR; nearly all (n=35) calculated the increase in cortisol following awakening (CARi) with only two pertaining to the total output of cortisol in the hour following awakening (CARg). Five effect sizes achieved statistical significance: A study of children at elevated risk of schizophrenia observed that the CARi was strongly associated with both current [*r=*0.52 (95% CI: 0.13 to 0.77)] and previous distress [*r=*0.51 (95% CI: 0.11 to 0.77)] in relation to negative life events in children with a FHx of schizophrenia, but found no significant associations in children presenting antecedents of illness (including PEs) or controls (31). Moreover, this study found that negative life event exposure, daily stressor exposure, and daily stressor distress were not associated with the CARi in any group. Similarly, no significant associations were found in any group between the CARi and life event exposure in three further studies that between them included controls, UHR individuals, and FEP patients (51, 65, 70). With regards to childhood trauma, significant associations were observed with the CARi in healthy controls that were not observed in FEP patients in two studies; however, in one study (46), the association in controls was negative [*r=*-0.43 (95% CI: -0.56 to -0.28)] whilst in the other study (70) the relationship was positive [*r=*0.39 (95% CI: 0.11 to 0.62)]. Interestingly, Ciufolini and colleagues also calculated the CARg and found a significant positive association in the control group [*r=*0.21 (95% CI: 0.04 to 0.37)] that was not present in the FEP group. In contrast, there were no significant associations in controls, UHR individuals, or FEP patients in any of three studies examining the relationship between perceived stress and the CARi (51, 65, 70).

#### Diurnal Cortisol

Thirty-nine effect sizes were available for diurnal cortisol, the majority of which (n=31) were AUCg values (i.e., the total cortisol output throughout the day), with the remaining (n=8) representing the diurnal slope (i.e., the decrease in cortisol from awakening to evening). Significant associations were found in a single study (65), in which life event exposure was negatively associated with diurnal AUCg cortisol in FEP patients [*r=*-0.36 (95% CI -0.58 to -0.09)] but positively associated in healthy controls [*r=*0.42 (95% CI: 0.11 to 0.66)]. In contrast, two further studies that assessed diurnal cortisol using the same sampling procedure as this study found no significant relationships with life event exposure or life event distress in any group (31, 51). Moreover, neither of the diurnal cortisol measures (AUCg or slope) were associated with childhood trauma, perceived stress, or daily stressor exposure/distress in any group (31, 46, 51, 52, 65, 70).

#### Hair Cortisol

We identified 38 individual effect sizes for hair cortisol, of which four were statistically significant. A single study (56) reported positive associations of hair cortisol with socioeconomic status [*r=*0.42 (95% CI: 0.08 to 0.67)] and lifetime trauma [*r=*0.48 (95% CI: 0.15 to 0.71)] among individuals with a FHx of illness, and similarly a positive association with lifetime trauma in individuals reporting PEs [*r=*0.31 (95% CI 0.01 to 0.56)]; none of these associations were significant in controls. However, this study observed no significant associations between hair cortisol and any other stressor (migration, minority status, perceived discrimination, social undermining, ostracism experience, bullying victimization, childhood abuse) in any group. The only other study to report a significant effect (55), found a negative relationship between childhood trauma and hair cortisol among patients with chronic schizophrenia [*r=*-0.66 (95% CI: -0.89 to 0.17)] that was not observed among patients with early schizophrenia, individuals at UHR, or healthy controls. In contrast, a further study of patients with established schizophrenia found no association between hair cortisol and childhood trauma (69); however, as childhood trauma data was not collected in controls, no comparison is available. Neither of the studies examining perceived stress reported significant associations in controls or psychosis spectrum groups (47, 54).

#### Experience Sampling Method (ESM) Cortisol

Two studies, yielding five effect sizes, assessed stressor-cortisol concordance using the ESM method. The first of these (60), reported that event stress was positively associated with cortisol in individuals with a FHx of psychosis [*B*=0.04 (95% CI: 0.00 to 0.08)], a relationship that was not present among healthy controls [*B*=0.00 (95% CI: -0.01 to 0.02)]. A later study by the same group (53) tested both linear and quadratic effects of event stress on cortisol, finding the latter to be a better fit. When using linear predictor terms, a significant positive association was observed among patients with psychotic disorder [*B*=0.28 (95% CI: 0.01 to 0.05)] that was not present among FHx individuals [*B*=-0.00 (95% CI: -0.02 to 0.02)] or controls [*B*=0.02 (95% CI: -0.04 to 0.01)]. Similarly, in the quadratic model a significant negative relationship (inverted U-shape) was detected in patients with psychotic disorder [*B*=-0.02 (95% CI: -0.03 to -0.00)] whereas positive (U-shaped), non-significant associations were found in the FHx [*B*=0.00 (95% CI: -0.01 to -0.02)] and control [*B*=0.12 (95% CI: -0.03 to 0.03)] groups.

### Meta-Analysis of Stressor-Cortisol Concordance

#### Overall Meta-Analysis of Stressor-Cortisol Concordance

**Figure 3:**
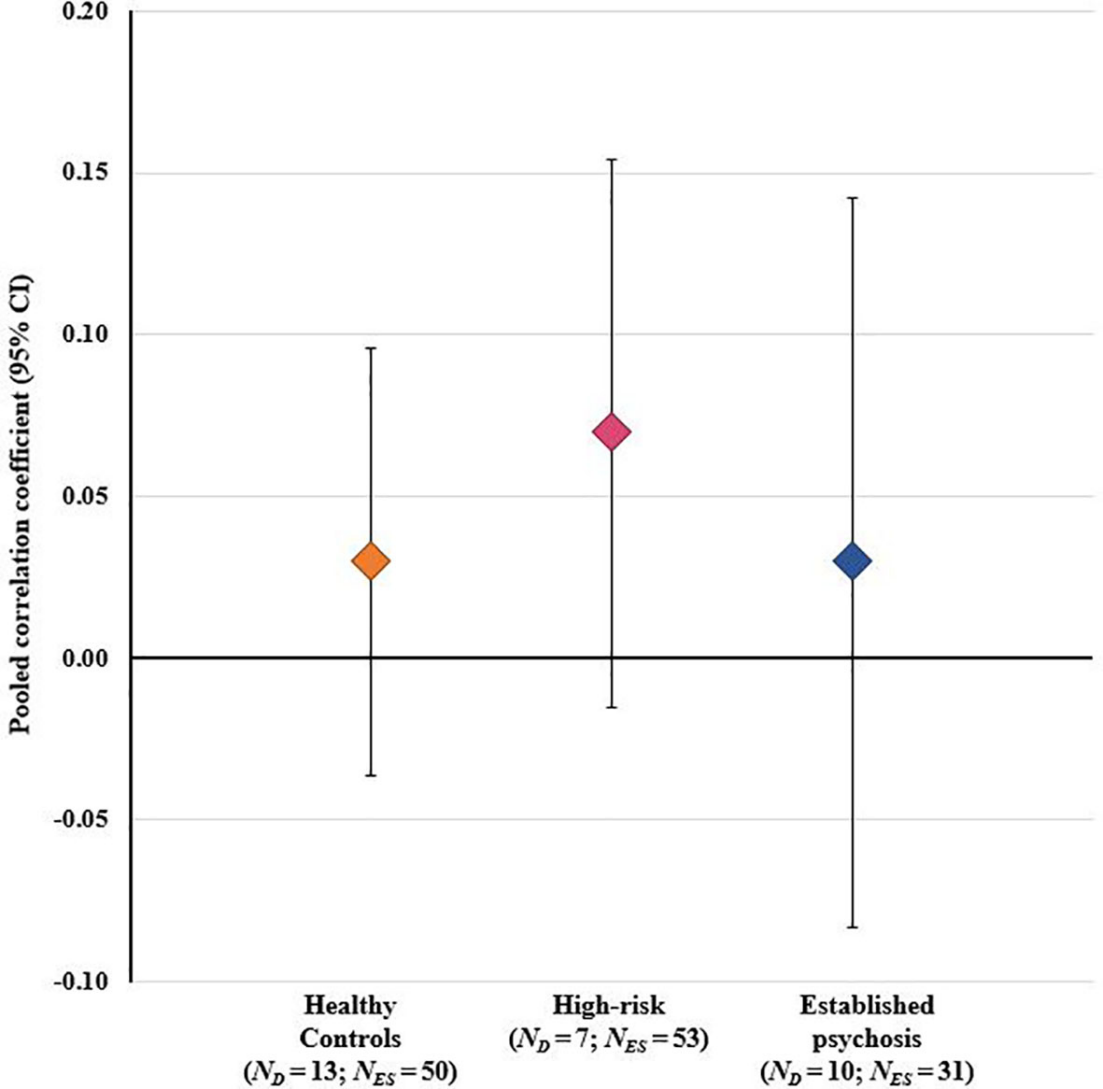
Results of overall meta-analysis comparing healthy controls, high-risk individuals, and patients with established psychosis on the degree of concordance between psychosocial stressors and cortisol across all stressor-cortisol pairings. CI, confidence interval; *N_D_*, number of study datasets contributing effect sizes; *N_ES_*, number of effect sizes included in pooled effect size.

The overall RVE model, which included data from all stressor-cortisol pairings, was performed on 134 effect sizes (beta coefficients were excluded as they could not be converted to a common metric). This model indicated a weak, positive association between stressors and cortisol that did not achieve statistical significance [*r=*0.05 (95% CI: -0.00 to 0.10), *p*=0.059]. A second model testing for group differences also showed no significant effect of either established psychosis status [*r=*0.01 (95% CI: -0.01 to 0.16), *p*=0.838] or high-risk status [*r=*0.02 (95% CI: -0.05 to 0.10), *p*=0.477] on effect sizes, indicating that the degree of concordance in these groups did not differ from healthy controls (see **Figure 3**). A further univariate regression model indicated no effect of study quality (NOS scores) on effect sizes [*r* = -0.01 (95% CI: -0.05 to 0.03), *p*=0.525]; moreover, as the funnel plot was not asymmetric (**Supplementary Figure 1**) there was no evidence of small sample bias. Heterogeneity estimates derived from the RVE model (τ^2^ = 0.016) indicated that 95% of the “true effects” were estimated to lie between *r* values of -0.20 and 0.30.

#### Stratified Analyses Examining Concordance Within Individual Stressor-Cortisol Pairings

**Figure 4:**
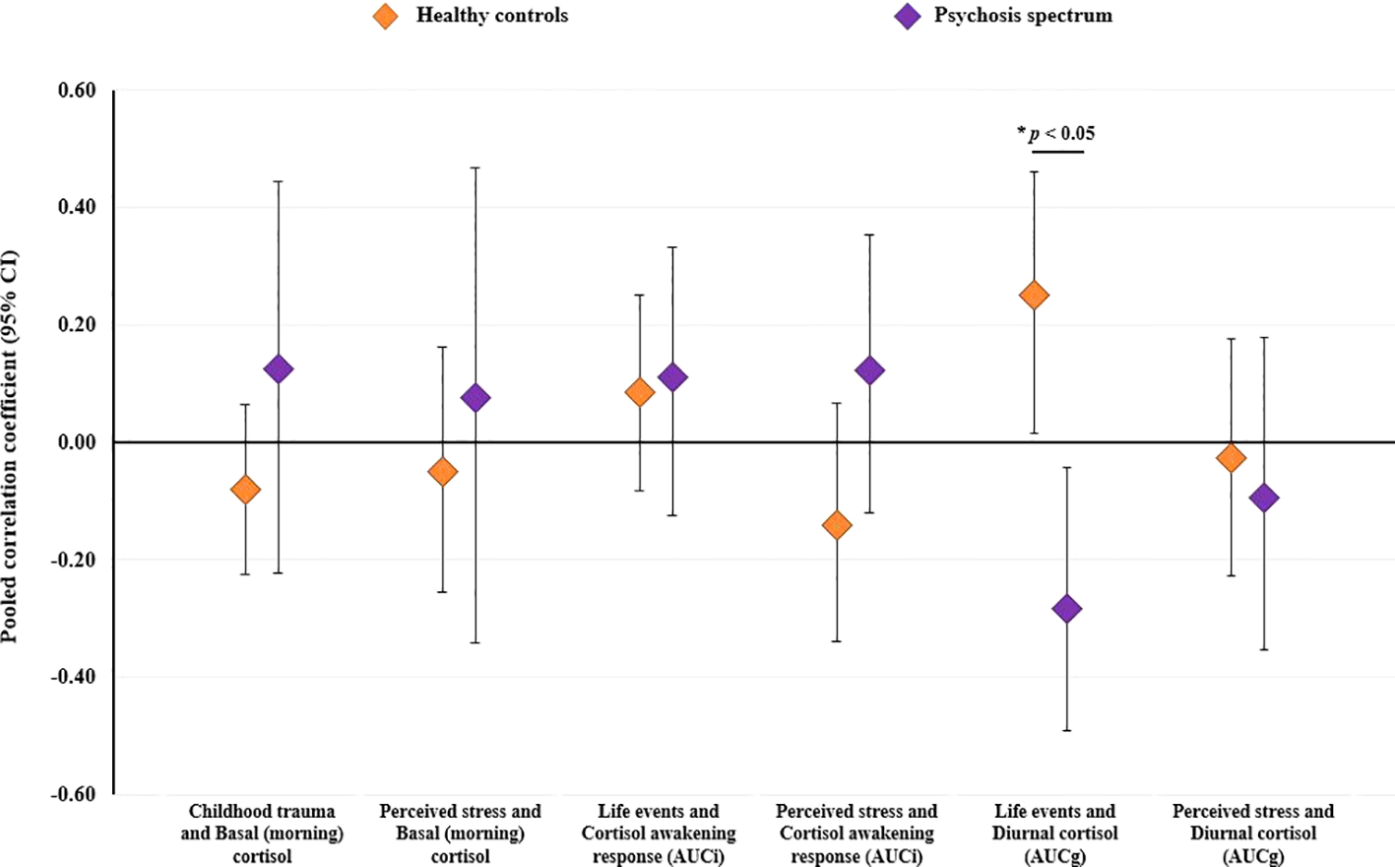
Results of stratified meta-analyses comparing healthy controls and individuals on the psychosis spectrum (established psychosis and high-risk groups combined) on the degree of concordance between psychosocial stressors and cortisol within individual stressor-cortisol pairings. CI, confidence interval.

Sufficient data were available to examine six individual stressor-cortisol pairings (i.e., these pairings were examined in three or more studies): i) childhood trauma and basal morning cortisol; ii) perceived stress and basal morning cortisol; iii) life event exposure and the CARi; iv) perceived stress and the CARi; v) life event exposure and diurnal cortisol (AUCg); and vi) perceived stress and diurnal cortisol (AUCg). Results of these stratified analyses are presented in **Table 3**. As illustrated in **Figure 4**, pooled effect sizes in both healthy control and psychosis spectrum groups were in the small-to-moderate range with both positive and negative associations observed. Statistically significant group differences were found for the association between life event exposure and diurnal cortisol (*p*=0.002); in controls a significant positive correlation was observed [*r=*0.25 (95% CI: 0.01 to 0.46)], whereas a significant negative correlation was observed in the psychosis spectrum group [*r=*-0.28 (95% CI: -0.49 to -0.04)]. No other group differences or individual effects achieved statistical significance. Overall, heterogeneity estimates ranged from low (particularly in the control group) to moderate, except for the association between childhood trauma and basal (morning) cortisol in the psychosis spectrum group, where substantial and significant heterogeneity was observed (I^2^ = 71%, *P* for Cochran’s Q=0.03).

**Table 3:** Subgroup meta-analyses comparing stressor-cortisol concordance in psychosis spectrum and healthy control groups.

Stressor-cortisol pairing	Datasets contributing to analysis	Healthy Controls					Psychosis Spectrum					HC vs. PS
		*N_ES_*	*r*	(95% CI)	*P* for Q	I^2^	*N_ES_*	*r*	(95% CI)	*P* for Q	I^2^	*P*
Childhood trauma & basal (morning)	Faravelli et al. (67); Labad et al. (70); Seidenfaden et al. (52)	3	-0.08	(-0.22–0.06)	0.93	0%	3	0.13	(-0.22–0.44)	0.03	71%	0.285
Perceived stress & basal (morning)	Garner et al. (68); Labad et al. (70); Seidenfaden et al. (52)	3	-0.05	(-0.26–0.16)	0.73	0%	4	0.07	(-0.34–0.47)	0.10	56%	0.611
Life events & CAR (AUCi)	Cullen et al. (2014); Labad et al. (70); Mondelli et al. (65); Nordholm et al. (51)	4	0.09	(-0.08–0.25)	0.85	0%	7	0.11	(-0.13–0.33)	0.96	0%	0.872
Perceived stress & CAR (AUCi)	Labad et al. (70); Mondelli et al. (65); Nordholm et al. (51)	3	-0.14	(-0.34–0.07)	0.36	0%	5	0.12	(-0.12–0.35)	0.62	0%	0.105
Life events & diurnal (AUCg)	Cullen et al. (2014); Mondelli et al. (65); Nordholm et al. (51)	**3**	**0.25**	**(0.01–0.46)**	**0.23**	**29%**	**5**	**-0.28**	**(-0.49–-0.04)**	**0.47**	**0%**	**0.002**
Perceived stress & diurnal (AUCg)	Mondelli et al. (65); Nordholm et al. (51); Seidenfaden et al. (52)	3	-0.03	(-0.23–0.18)	0.57	0%	4	-0.09	(-0.35–0.18)	0.25	34%	0.698

CAR, cortisol awakening response; AUCi, area-under-the-curve with respect to increase; AUCg, area-under-the-curve with respect to ground; N_ES_, total number of effect sizes included before within-study pooling; CI, confidence interval; P for Q, P value associated with Cochran’s Q; HC, healthy control; PS, psychosis spectrum.

Bold text indicates that the effect size comparison between HC and PS groups is statistically significant at P<0.05.

## Discussion

In the first meta-analysis to compare associations between naturally-occurring psychosocial stressors and cortisol in individuals on the psychosis spectrum and healthy controls, we observed poor concordance irrespective of stressor type, cortisol measure, or group status. The overall model, comprising 134 effect sizes, showed that stressors and cortisol measures were only weakly (and not significantly) correlated. Moreover, meta-regression analyses indicated that effect sizes among individuals with established psychosis and those at high-risk for psychosis did not differ from controls. In stratified analyses, performed to test for group differences within individual stressor-cortisol pairings, significant differences between healthy controls and psychosis spectrum groups were observed for only one of the six stressor-cortisol pairings examined. Thus, we found little evidence to suggest that cortisol responses to naturally-occurring stressors are any different in individuals on the psychosis spectrum compared to healthy controls.

Of the four *a priori* alternative hypotheses presented (**Figure 1**), our findings are most consistent with the “unrelated” hypothesis: Regardless of whether analyses were conducted using all effect sizes (excluding those derived from ESM studies which could not be pooled), or within individual stressor-cortisol pairings, the degree of concordance was weak, and, in most instances, did not differ across groups. The only significant group difference that we observed was for the relationship between life events and diurnal cortisol where significant associations were found in both controls and psychosis spectrum groups, but the direction of these effects differed (a positive correlation was observed in controls and negative correlation in the psychosis spectrum group). However, this finding appeared to be driven by a single study (65) which reported significant, opposing relationships between life events and diurnal cortisol in FEP patients and controls. Indeed, two subsequent studies which used the same protocol for obtaining cortisol samples in the home environment as Mondelli and colleagues found no significant associations between life events and diurnal cortisol in any group (31, 51), which is particularly surprising given that that the latter employed the same life event measure and included FEP patients (51). Our findings contrast with those observed in studies examining cortisol responses to acute psychosocial stressor tasks, in which healthy controls show a robust cortisol response whilst patients with psychosis and schizophrenia (37–39) and those at high-risk for psychosis (74, 75) demonstrate a blunted cortisol response. Together, these findings suggest that the cortisol abnormalities previously observed among psychosis spectrum groups (e.g., elevated basal and diurnal cortisol and a blunted CAR) are unlikely to be driven by greater exposure and/or sensitivity to these psychosocial stressors (which do not appear to elicit a robust cortisol response). Instead, these HPA axis abnormalities might be epiphenomenal, perhaps secondary to medication effects, substance use, or a manifestation of global physiological dysregulation. Moreover, these findings suggest that psychosocial stressors may contribute to the onset and exacerbation of psychotic illness *via* other mechanisms (e.g., cognitive processes, or the immuno-inflammatory system) as opposed to cortisol fluctuations.

There are several reasons why we should be cautious about drawing these conclusions; these fall into the broad domains of statistical power, analytical approaches, heterogeneity (relating to study measures and populations), and timing of cortisol collection in relation to stressor onset/measurement, which will now be discussed in turn. First, low statistical power at both the study level and meta-analysis level may explain the poor concordance we observed between stressors and cortisol. Our systematic review indicated that only a small number of studies (2/18) included sufficient participants in each group to be able to detect a moderate correlation. Given that the majority of studies (87%) reported negligible-to-small correlations, this might explain why so few statistically significant correlations were observed at the study level. At the meta-analysis level, we were only able to include data from 16 separate datasets, far less than the minimum number (N=40) recommended (63). As such, our meta-analyses were almost certainly underpowered.

The analytic approaches adopted across studies may have also contributed to poor concordance. With regards to the analysis of cortisol data, a study of healthy females reported that while perceived stress scores showed no relationship with absolute cortisol levels (mean of multiple samples obtained throughout a single day), they were significantly associated with change in cortisol levels (76), implying that cortisol measures indexing deviation from normal HPA axis activity may be more sensitive to psychosocial stress. Indeed, this might explain why studies employing acute psychosocial stressor tasks observe a robust “stressor effect” (i.e., an increase in cortisol from baseline as a result of task anticipation and commencement). However, it should be noted that we did not observe this pattern in our review: In fact, more than half of the individual effect sizes that achieved statistical significance (10/16) pertained to absolute measures of cortisol (i.e., basal levels, diurnal AUCg, hair cortisol). Another analytical issue pertains to adjustment for potentially confounding factors, which substantially varied across studies. Cortisol levels have been associated with a range of participant factors, including age, sex, ethnicity, socioeconomic status, and psychotropic medication, factors which often distinguish psychosis spectrum and healthy control groups (20). As such, failure to account for these factors may mask important group differences.

Heterogeneity across studies with regards to study measures may have impacted on our ability to detect a significant overall association between psychosocial stressors and cortisol. In this review, we examined a broad range of psychosocial stressors, including: exposure to specific, pre-defined events (daily stressors, recent and major life events, childhood trauma); distress related to these specific events; subjectively-rated stressfulness of current activities (ESM activity stress); and appraisals of the degree to which life is stressful, unpredictable, and uncontrollable (perceived stress). While all of these measures are relevant to the concept of “stress” (either because they index events that most individuals would consider to be stressful, or because they capture subjective experiences of stress/distress) there is likely substantial variability in the extent to which they are associated with a biological stress response. Moreover, perceived stress has been found to correlate with both personality traits and depressive symptoms (77), suggesting that it can be considered a trait-like feature rather than a measure of stress exposure per se. Indeed, this might explain why perceived stress was not associated with cortisol in any of the studies included in our review. Coupled with the fact that, as noted above, cortisol measures also varied substantially across studies, it is perhaps unsurprising that we observed substantial heterogeneity in effect sizes across studies. In addition to this, heterogeneity in the study populations examined may have contributed to our inability to detect significant differences between individuals on the psychosis spectrum and healthy controls. In our overall analysis, we were able to differentiate between individuals with established psychosis and those at high-risk for the disorder; however, even within these subcategories there was substantial variability. The established psychosis group included patients with diagnoses of first-episode psychosis, early stage schizophrenia, and chronic schizophrenia who likely differed with regards to exposure to antipsychotic medication and other confounding factors known to influence cortisol levels (20). However, there was perhaps even greater variability within the high-risk groups, which included help-seeking individuals meeting UHR criteria (who present features consistent with the prodromal phase of psychosis); adolescents and adults with a family history of illness; and individuals reporting PEs. Within these groups, the proportion of individuals who will go on to develop full psychosis varies considerably (45, 78); indeed, it is likely that FHx individuals who reach adulthood without developing psychosis do so due to protective factors. In our review, we chose to include populations that are frequently defined in the literature as being at “high-risk” for psychosis on the basis that the neural diathesis-stress model describes a mechanism that may operate in those with increased vulnerability for psychosis, irrespective of cause (18, 20). Nevertheless, it is important to note that this may have contributed to substantial heterogeneity in effect sizes across studies.

A further possible explanation for the poor concordance we observed is that cortisol samples are unlikely to have been collected at the time of stressor exposure. A previous meta-analysis (41) found that the degree of concordance between chronic stress and cortisol is strongly influenced by the lapse-of-time between stressor exposure and cortisol measurement (i.e., as time since stressor onset increases, the degree of concordance diminishes). However, this pattern did not emerge in the present review; rather, significant associations with cortisol were observed for both distal (e.g., childhood trauma) and proximal events (e.g., ESM event stress). As a related issue, it is possible that the time-lapse between stress *measurement* and cortisol collection might be a contributing factor. A recent study using data from a large sample of individuals at UHR for psychosis and healthy controls indicated that the degree of concordance between psychosocial stressors and basal cortisol was moderated by the lapse-of-time between collection of these measures (79): Specifically, daily stressors, life events, and childhood trauma, were only associated with basal cortisol measures when these stress measures were completed on the same day as cortisol collection. The fact that this pattern was observed for daily stressors occurring within the last 24-h and life events/childhood trauma (which did not occur on the day of testing) suggests that distress associated with recalling these events might elicit a cortisol response that enables a significant association to be observed. Importantly, after accounting for the lapse-of-time between assessments, analyses indicated that the degree of concordance was stronger among CHR individuals who later converted to psychosis when compared to those who did not (79); thus, accounting for the time-lapse between assessments may improve precision and reveal important group differences. In the present review, we found that only three studies reported the lapse-of-time between stress measurement and cortisol collection, and only two confirmed that measures were completed on the same day. Both of these studies used the ESM approach to obtain cortisol samples within 10 min of stressor ratings (53, 60); however, even with this short lapse-of-time, significant associations between event stress and salivary cortisol were not observed in healthy controls, only those on the psychosis spectrum (i.e., relatives and patients with psychotic disorders). Interestingly, a recent ESM study examining individuals with 22q11.2 deletion syndrome (a syndrome associated with learning difficulties, a range of physical health problems, and psychiatric comorbidity—including psychosis), reported that cortisol levels in the healthy control group, but not the 22q11.2 deletion syndrome group, increased in parallel with activity related stress, but that this association in controls was only significant at the trend level (80). Together, these findings suggest that the activity-/event-related stress captured using existing ESM approaches may not be sufficiently “stressful” to elicit robust changes in cortisol levels in healthy controls.

In summary, there are a number of important methodological issues that contribute to complexity when examining the relationship between psychosocial stressors encountered in the natural environment and cortisol. While none of these potential explanations can fully account for the poor concordance that we observed across a range of stressor and cortisol measures, it is certainly possible that methodological issues obscured the ability to detect “true” associations between these measures.

### Limitations

As noted above, given that we were only able to include data from 18 studies (representing 16 independent datasets) our meta-analyses were likely underpowered. This would have affected our ability to detect statistically significant correlations between stressors and cortisol (which were largely within the small-to-moderate range), and to test for group differences in the degree of concordance. However, it is important to note that previous meta-analyses have observed group differences in cortisol responses to psychosocial stressor tasks with far fewer studies (37, 39). The small number of studies identified also meant that in our stratified analyses (testing group differences within individual stressor-cortisol pairings) it was necessary to combine effect sizes derived from patients with established psychosis and individuals at high-risk for psychosis in a single “psychosis spectrum” group. As such, the psychosis spectrum group was highly heterogeneous. It is possible that the inclusion of individuals at different stages of illness (from adolescents reporting isolated psychotic experiences to adult patients with chronic schizophrenia) with varying degrees of psychopathology may have diluted any group differences; indeed, recent theories propose that different stages of illness may be associated with different patterns of HPA axis dysregulation (35). However, in our overall analysis (which included all effect sizes) we were able to distinguish between established psychosis patients and high-risk participants and found no substantial difference in effect sizes in these groups. As noted above, there was also substantial heterogeneity across studies with regards to both psychosocial stressors (ranging from minor daily stressors to major life events and childhood trauma) and cortisol measures (which included both dynamic measures such as the CAR, and chronic cortisol levels as measured in hair samples). This was reflected in the heterogeneity estimates derived from the overall model where the interval within which 95% of the “true effects” were estimated to lie was wide and crossed zero (-0.20 to 0.30). While this questions the extent to which these effect sizes could be pooled using meta-analytic techniques, we performed stratified analyses to reduce this heterogeneity. Moreover, pooling these results enabled us to quantify the level of heterogeneity and address the key question of whether the strength of association, irrespective of stressor-cortisol type, differed in healthy controls and those on the psychosis spectrum. A further limitation, noted above, pertains to the fact that our search was restricted to studies examining cortisol, as such, we did not consider other potential markers of HPA axis function (e.g., adrenocorticotropic hormone, hippocampal/pituitary volume, or glucocorticoid receptor density, distribution and/or affinity). However, cortisol is one of the most widely used indicators of HPA axis function and expanding our search parameters would have likely yielded an unmanageable number of studies to assess for eligibility. These limitations are balanced by several strengths. First, we employed robust statistical approaches to account for dependence of effect sizes, thereby allowing us to include multiple effect sizes from the same study. Second, to avoid potential cancelling effects (i.e., deriving a neutral effect by combining positive and negative associations) we additionally conducted stratified analyses where effect sizes were pooled within individual stressor-cortisol pairings (although the number of studies contributing to each analysis was substantially reduced). Finally, we included a wide range of psychosocial stressors and cortisol measures, increasing the number of studies in the review.

### Implications

As noted above, there are several methodological issues that might explain the poor concordance that we observed between psychosocial stressors and cortisol. As such, we recommend that future studies in this field i) conduct *a priori* power calculations to determine the minimum number of participants required for each group and ensure that recruitment is matched to the target number; ii) investigate within-subject deviation from normative cortisol levels, whether this be daily fluctuations (i.e., increase from awakening or other time-point) or variations across days (i.e., changes from mean level), as these variations may be more strongly associated with psychosocial stressors; iii) move beyond simple cross-sectional analyses and instead attempt to obtain longitudinal measures of both stressors and cortisol in order to disentangle the temporal relationship between these measures; iv) report the lapse-of-time between stressor assessment and cortisol collection and test whether this variable moderates the strength of association (and, if so, account for interaction effects accordingly); and (v) investigate potential confounders and adjust analyses as appropriate. It is important to note that we found no association between study quality/bias scores (which considered some of these factors) and effect sizes. As such, it is possible that these recommendations will not necessarily increase the likelihood that a study is able to detect concordance between naturally-occurring psychosocial stressors and cortisol; however, this is an important first step to elucidating these relationships.

Our findings should be considered with reference to existing theories of psychosis aetiology. The neural diathesis-stress model of schizophrenia hypothesized that HPA axis dysregulation among those on the psychosis spectrum could be stress-induced, a manifestation of hippocampal dysfunction or glucocorticoid receptor abnormalities, or genetically determined (18–20). While the current review provides no evidence to suggest that cortisol abnormalities among individuals on the psychosis spectrum are stress-induced, we again emphasize the need to consider the range of methodological issues that might have contributed to this null finding. Aside from the aforementioned methodological issues, it is also possible that repeated exposure to psychosocial stressors among individuals on the psychosis spectrum leads to an initial increase in HPA axis function, that, when exhausted, leads to a dysregulated system that no longer responds to stress appropriately—as proposed in the tonic/phasic model of HPA axis dysregulation (35). However, this would not explain why healthy controls (who we know experience lower levels of psychosocial stressor exposure and distress) also showed poor concordance, and we would have also expected to see variability in the degree of concordance across illness phases had this been the case. Our review provides important findings regarding the relationship (or lack of) between psychosocial stressors and cortisol that should be incorporated in future revisions to these theories.

### Conclusions

This comprehensive systematic review and meta-analysis found no evidence to suggest that individuals on the psychosis spectrum are characterized by either hyper- or hypo-responsivity of the HPA axis to naturally-occurring psychosocial stressors. These findings are in contrast to the blunted cortisol response observed during psychosocial stressor tasks among patients with established illness and individuals at high-risk for psychosis. While our findings suggest that psychosocial stressors cannot explain the cortisol abnormalities that have been previously reported in psychosis spectrum groups, this might also reflect methodological issues that are common to studies of naturally-occurring psychosocial stressors (e.g., failure to acquire cortisol samples proximal to stress exposure/assessment) but are tightly controlled in experimental studies employing psychosocial stressor tasks. Moreover, without adequate assessment of potential confounders and moderating factors, no conclusions can be drawn regarding the true relationship between psychosocial stressors encountered in the natural environment and cortisol levels. Thus, we strongly advocate that future studies attempting to investigate stressor-cortisol concordance consider these factors during the study planning phase and when conducting analyses. Nevertheless, the current evidence suggests that cortisol responses to naturally-occurring stressors are not a robust marker of either risk for psychosis or established illness.

## Data Availability Statement

The dataset generated and analyzed in this study is available from AC on request.

## Author Contributions

AC conceived the study, oversaw all systematic searches, conducted all statistical analyses, and wrote the first draft of the manuscript. SR and MV contributed equally to the study and were responsible for conducting the systematic search, reviewing studies for eligibility, extracting study characteristic data, and rating study quality/bias. VM and PM contributed intellectually to the critical interpretation of results. All authors reviewed and contributed to the final manuscript.

## Funding

AC is supported by a Sir Henry Welcome Postdoctoral Fellowship from the Wellcome Trust (107395/Z/15/Z), and a NARSAD Young Investigator Grant awarded by the Brain & Behavior Research Foundation (28336) and funded by the Evelyn Toll Family Foundation. This paper represents independent research part-funded by the National Institute for Health Research (NIHR) Biomedical Research Centre at South London and Maudsley NHS Foundation Trust and King’s College London (IS-BRC-1215-20018 awarded to AC). The views expressed are those of the authors and not necessarily those of the NHS, the NIHR or the Department of Health and Social Care. The funders had no role in the design and conduct of the study; collection, management, analysis, and interpretation of the data; preparation, review, or approval of the manuscript; or decision to submit the manuscript for publication.

## Conflict of Interest

The authors declare that the research was conducted in the absence of any commercial or financial relationships that could be construed as a potential conflict of interest.

The handling editor declared a past co-authorship with one of the authors, PM.
