# Supplementary figures and images for: Cortisol Responses to Naturally Occurring Psychosocial Stressors Across the Psychosis Spectrum: A Systematic Review and Meta-Analysis
Source: Front Psychiatry. 2020 Jun 11;11:513. doi: 10.3389/fpsyt.2020.00513 (PMC7300294; doi:10.3389/fpsyt.2020.00513)

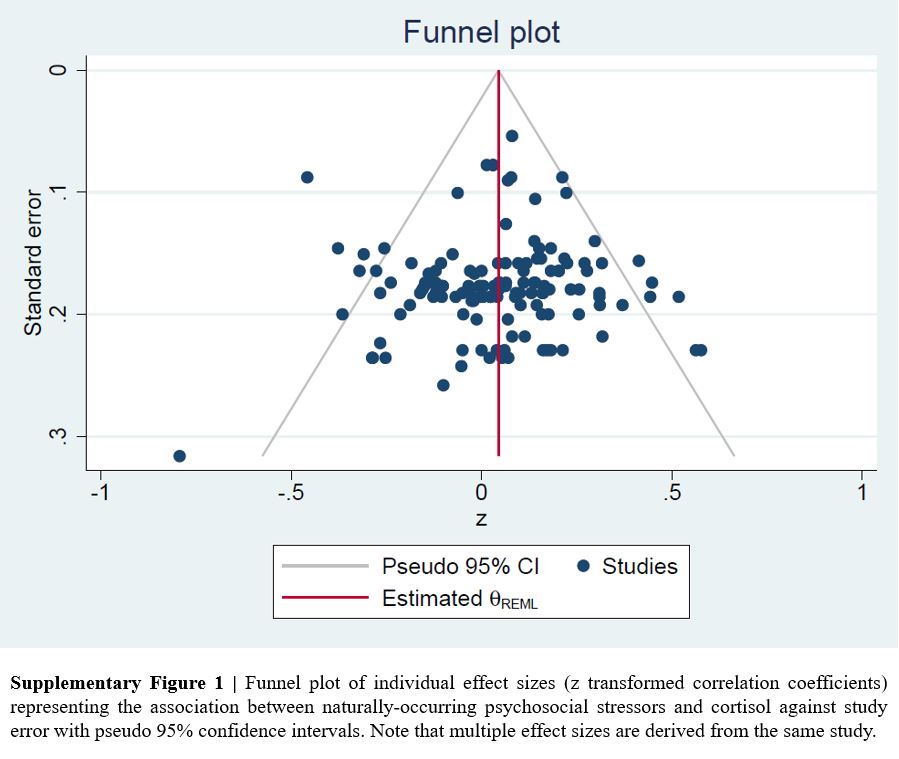

Supplement: Supplementary file 1 [file Image_1.jpeg]
