# Supplementary material for: Cortisol Responses to Naturally Occurring Psychosocial Stressors Across the Psychosis Spectrum: A Systematic Review and Meta-Analysis
Source: Front Psychiatry. 2020 Jun 11;11:513. doi: 10.3389/fpsyt.2020.00513 (PMC7300294; doi:10.3389/fpsyt.2020.00513)
Supplement: Supplementary file 2 [file Table_1.docx]

Supplementary Material

**Supplementary Table 1 |** Modified Newcastle-Ottawa scale items used to rate study quality

| **Item (max score)** | **Description** |
| --- | --- |
| Sample size adequate / determined a priori (max 2) | Ideally, calculated *a priori* by study authors to provide sufficient power to detect association between stressor and cortisol, where target group size is achieved for all groups 2 points can be awarded. If no sample size calculation performed, all groups should of sufficient size (n≥85) needed to detect a moderate effect size (r=0.30 at 80% power) to obtain 1 point. |
| Psychosis spectrum definition valid  (max 1) | Definition appropriate to psychosis spectrum group, for example PACE criteria using valid tool (e.g., CAARMS/SIPS) for UHR, family history ascertained using structured interview (e.g., FIGS) for FHx, or, diagnosis confirmed using a structured interview (e.g., SCID) for established psychosis. Score of 1 obtained when *every* psychosis spectrum group defined adequately. |
| Psychosis spectrum cases unbiased (max 1) | Psychosis spectrum group should be representative of the target population that the authors wish to generalize to (e.g., randomly selected from register including all patients within a catchment area). 1 point awarded for representative samples with low risk of bias. |
| Control group unbiased (max 1) | Healthy control group should be representative of the general population within given country/state. Representativeness will depend on sex, age, ethnicity, socioeconomic status, occupation etc. Efforts should be made to ensure healthy control group are not biased (i.e., that levels of stress or HPA axis functioning should not influence extent to which individual is selected as a control). Studies score 1 point where there is no evidence to suggest control group are unrepresentative or biased. |
| Control status confirmed (max 1) | Efforts made to ensure healthy controls do not meet criteria for psychosis spectrum group. 1 point awarded when the same measures to determine psychosis spectrum status are also be administered to controls. |
| Response rate reported and same in both groups (max 1) | The response rate (i.e., proportion of individuals who agreed to participate out of the total number of individuals invited to participate in the study) should be reported; ideally comparisons should be made on demographic characteristics between those who did and did not agree to participate. Studies can be awarded a point if response rates are reported for both psychosis spectrum and control groups. |
| Psychosis spectrum and control groups matched (max 2) | Psychosis spectrum and control groups should be matched on key factors likely to influence psychosocial stress and cortisol levels (e.g., age and sex). Studies that perform matching at the point of recruitment (on both age and sex) should receive 2 points. Where matching is determined post-hoc (i.e., analyses are conducted to show that groups do not differ on age and sex) 1 point can be awarded. |
| Stress measure reliable / valid  (max 2) | Study should employ use well-established tools to measure stress and report reliability for the measure and the time-frame assessed. For 2 points it should report the reliability and say whether it has been validated for the sample. For 1 point it would be a well-established measure (no reliability reported). If authors developed their own tool, 2 points can be awarded if tests performed to establish reliability and/or validity in the sample. |
| Cortisol measure reliable / valid  (max 2) | Study should use a robust procedure for collecting cortisol samples with appropriate details provided (i.e., time of collection, storage and analysis methods) to achieve 2 points. Diurnal and CAR protocols should involve a minimum of two samples. Studies utilizing home collection protocols should report steps to monitor compliance with the procedure. 1 point can be awarded for studies that provide partial fulfilment of criteria. |
| Lapse of time between measures reported (max 1) | Authors should report the lapse of time between measurement of psychosocial stressors and collection of cortisol samples (e.g., whether both measures collected on the same day) for 1 point. |
| Potential confounds examined (max 2) | Study should ideally measure a range of potential confounders that might affect stressor-cortisol concordance (e.g., medication, tobacco use, BMI) and conduct statistical analyses to compare groups on these measures and/or assess the influence of these factors on stress/cortisol. 2 points awarded for studies that measure multiple potential confounders and compare groups on these factors; 1 point can be awarded where a single potential confounder measured, or multiple potential confounders are measured but no statistical tests are performed. |

PACE: Personal Assessment and Crisis Evaluation (Yung et al., 2007); CAARMS: Comprehensive Assessment of the At-Risk Mental States (Yung et al., 2005); SIPS: Structured Interview for Prodromal Syndromes (McGlashan et al., 2010); UHR: ultra-high risk; FIGS: Family Interview for Genetic Studies (Maxwell, 1992); FHx: family history of psychosis; SCID: Structured Clinical Interview (First et al., 1995); CAR: cortisol awakening response; BMI: body mass index.

**References**

First, M., Spitzer, R.L., Gibbon, M., Williams, B., and Williams, J.B.W. (1995). *Structured Clinical Interview for DSM-IV Axis I Disorders, Patient Edition.* New York: Biometrics Research Department, New York State Psychiatric Institute, New York.

Maxwell, M.E. (1992). *Family Interview for Genetic Studies.*: National Institute of Mental Health: St. Louis, MO.

Mcglashan, T., Walsh, B.C., and Woods, S.W. (2010). *The Psychosis-Risk Syndrome: Handbook for Diagnosis and Follow-up.* New York, NY:: Oxford University Press Inc.

Yung, A.R., Mcgorry, P.D., Francey, S.M., Nelson, B., Baker, K., Phillips, L.J., Berger, G., and Amminger, G.P. (2007). PACE: a specialised service for young people at risk of psychotic disorders. *Med J Aust* 187**,** S43-46.

Yung, A.R., Yuen, H.P., Mcgorry, P.D., Phillips, L.J., Kelly, D., Dell'olio, M., Francey, S.M., Cosgrave, E.M., Killackey, E., Stanford, C., Godfrey, K., and Buckby, J. (2005). Mapping the onset of psychosis: the Comprehensive Assessment of At-Risk Mental States. *The Australian and New Zealand Journal of Psychiatry* 39**,** 964-971.
